# Deep Learning-Based Industry 4.0 and Internet of Things towards Effective Energy Management for Smart Buildings

**DOI:** 10.3390/s21041038

**Published:** 2021-02-03

**Authors:** Mahmoud Elsisi, Minh-Quang Tran, Karar Mahmoud, Matti Lehtonen, Mohamed M. F. Darwish

**Affiliations:** 1 Industry 4.0 Implementation Center, Center for Cyber–Physical System Innovation, National Taiwan University of Science and Technology, Taipei 10607, Taiwan; mahmoud.elsisi@mail.ntust.edu.tw (M.E.); minhquang.tran@mail.ntust.edu.tw (M.-Q.T.); 2 Department of Electrical Engineering, Faculty of Engineering at Shoubra, Benha University, Cairo 11629, Egypt; 3 Department of Mechanical Engineering, Thai Nguyen University of Technology, 3/2 Street, Tich Luong Ward, Thai Nguyen 250000, Vietnam; 4 Department of Electrical Engineering and Automation, Aalto University, FI-00076 Espoo, Finland; karar.mostafa@aalto.fi (K.M.); matti.lehtonen@aalto.fi (M.L.); 5 Department of Electrical Engineering, Faculty of Engineering, Aswan University, Aswan 81542, Egypt

**Keywords:** smart systems, internet of things, machine learning, energy management

## Abstract

Worldwide, energy consumption and saving represent the main challenges for all sectors, most importantly in industrial and domestic sectors. The internet of things (IoT) is a new technology that establishes the core of Industry 4.0. The IoT enables the sharing of signals between devices and machines via the internet. Besides, the IoT system enables the utilization of artificial intelligence (AI) techniques to manage and control the signals between different machines based on intelligence decisions. The paper’s innovation is to introduce a deep learning and IoT based approach to control the operation of air conditioners in order to reduce energy consumption. To achieve such an ambitious target, we have proposed a deep learning-based people detection system utilizing the YOLOv3 algorithm to count the number of persons in a specific area. Accordingly, the operation of the air conditioners could be optimally managed in a smart building. Furthermore, the number of persons and the status of the air conditioners are published via the internet to the dashboard of the IoT platform. The proposed system enhances decision making about energy consumption. To affirm the efficacy and effectiveness of the proposed approach, intensive test scenarios are simulated in a specific smart building considering the existence of air conditioners. The simulation results emphasize that the proposed deep learning-based recognition algorithm can accurately detect the number of persons in the specified area, thanks to its ability to model highly non-linear relationships in data. The detection status can also be successfully published on the dashboard of the IoT platform. Another vital application of the proposed promising approach is in the remote management of diverse controllable devices.

## 1. Introduction

Nowadays, urbanization is increasing rapidly, particularly in developed countries, leading to a high demand for energy consumption. According to the International Energy Agency, from 1971 to 2014, worldwide energy consumption has grown by 92%. About 80% of global energy is consumed by the urban agglomerates, of which the total energy consumption of buildings accounts for 40% [1]. This is one of the reasons that we are facing pollution and climate change issues. Electricity is derived from different sources such as hydropower, nuclear fission reactions, natural gas, coal, wind, and sunlight, which are limited energy resources. However, it is being used wastefully because of user habits like forgetting to turn off electrical devices, not knowing how to use them, or using devices that consume too much power. The energy efficiency in buildings is vital for the environment and global sustainability [2]. Therefore, energy consumption and saving are a great concern for our current time, and the internet of things (IoT)-based energy management of smart buildings is a particularly important research topic.

The IoT is a new technology that establishes the core of Industry 4.0. The IoT system consists of many emerging technologies that enable wireless interconnections among physical objects, which, in this system, are referred to as “things”. The data-gathering sensors are equipped to IoT devices such as appliances, personal devices, or industrial equipment that allow communication and control. It is predicted that the number of smart devices connected to the IoT will be around 30 billion worldwide by 2020 [3]. With the evolution of IoT topology, the online data obtained from the sensors can be utilized to improve the industrial processes and the quality of life [4]. Therefore, this technology is recognized as one of the most remarkable technological advances in future technology and received great attention due to its potential in empowering the fourth industrial revolution [5].

There are several IoT-related technologies that have been developed to monitor energy consumption and energy saving [4,6,7]. The internet of things can provide a solution for building smart energy control systems of buildings, particularly for smart city applications [8]. It is reported that roughly 50% of buildings’ energy is consumed by the residential sector, including cooking, lighting, heating, fanes, and air conditioning (HVAC). Thus, HVAC control systems represent an important effort in reducing electricity consumption [9]. An efficient IoT architecture based smart home was proposed for intelligent energy-saving [7]. In this example, the lights and the heating/cooling system could be controlled using light and HVAC control systems coupled with a surveillance camera, including a motion detection to remotely switch on/off when an occupant exits or enters the home. An intelligent energy management model that is aware of the electricity consumption in real-time through web and mobile devices has been introduced to remotely control electricity usage for homes and buildings [10]. The building energy consumption can be reduced significantly through correct detection of occupancy. This approach presents a great opportunity for energy savings [11]. A low-cost sensor platform was designed and implemented to be used for occupancy detection in individual offices [12]. Although the estimation of the occupancy count can further save the energy of the buildings, this requires a system that can accurately detect and give information about the occupancy. A passive infrared (PIR) sensor, usually used to measure the infrared light radiating from objects, has been widely employed to detect human movement and presence [11,13,14]. However, the displacement of the body from the PIR sensor, the speed, and the movement direction, reflects the analog output waves of PIR sensors. As a result, the PIR sensors are unable to accurately detect the number of people in a space, particularly with stationary human subjects [15,16].

Accordingly, it is important to incorporate more advanced machine learning techniques to successfully collect, manage, and analyze data in real-time because of the restricted capabilities of the IoT devices for smart buildings. With the development of machine learning, deep learning is recognized as an effective tool that is able to achieve end-to-end learning patterns and highly non-linear relationships in data. The deeper architectures of deep learning technologies allow them to learn more complicated features than shallow ones. Their reliable training algorithms are capable of learning informative target features without the need to extract and choose the important features manually [17]. There is no doubt that deep learning was an excellent strategy for dealing with a nonlinear issue of stationary human recognition. The IoT platform with a machine learning-based energy monitoring system is a promising approach for monitoring smart buildings to reduce energy consumption and simultaneously remain comfort and safety [18]. In ref. [19], You Look Only Once (YOLO-v3) is reported as one of the best algorithms for object detection among deep learning techniques [19]. This model has been developed to increase the recognition speed in offline and real-time situations. However, objects appearing too close in the image, or the detection of small objects, represent some drawbacks of this algorithm [20]. The demonstration of YOLO by utilizing the Darknet deep learning library can cope with recognition issues. Furthermore, it achieves a state of the art for real-time object detection. Since the YOLO network can recognize targets in the image without the requirement of the region proposal network by directly performing regression, this allows YOLO to perform much faster detection. Recently, the state-of-art version (YOLOv3) has been used to attain higher precision, accuracy and speed and optimize for the detection of small targets [21]. The original YOLOv3 network optimized its anchors for the head tracking part; the detection accuracy of passenger flow density in a metro system reached 95% [22].

**Figure 1:**
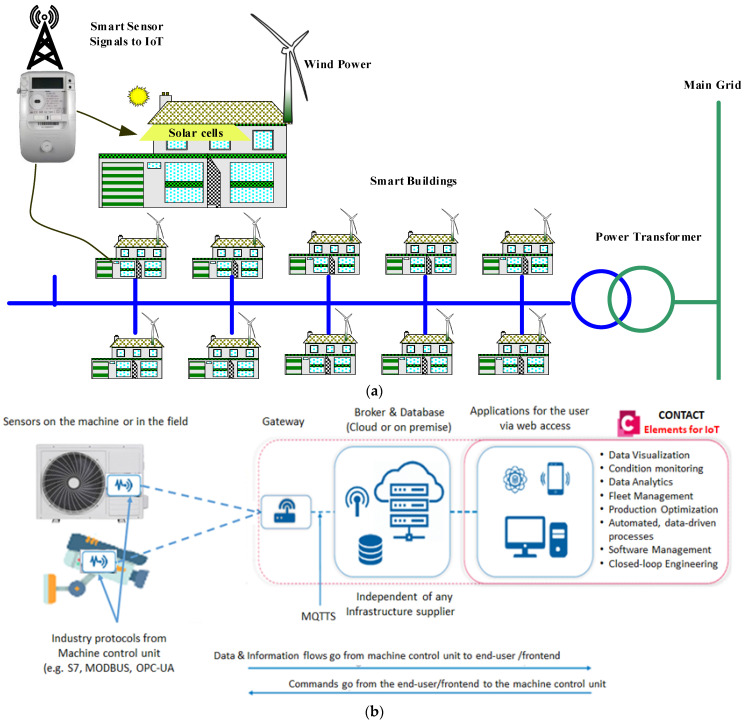
System diagram of the IoT based architecture: (**a**) smart buildings in electrical power systems, (**b**) air conditioner control unit connected to the IoT platform.

The fourth industrial revolution (i.e., Industry 4.0) and industrial IoT based on deep learning or big data are quickly driving data and software solutions driven digitalization in numerous areas. Inspired by the development of IoT and machine learning technology toward energy saving, a deep learning-based people detection system is proposed to count the number of persons in a typical smart building, making it possible to optimally manage the operation of the existing air conditioners. Specifically, an algorithm utilizing YOLOv3, that can remotely control the devices, is developed based on the number of persons and the status of the air conditioners via the IoT platform. The proposed topology can enhance the decision making about energy consumption by publishing the number of persons and the status of the air conditioners via the internet to the dashboard of the IoT platform. The proposed deep learning-based recognition algorithm with its ability to model highly non-linear relationships can accurately detect the number of persons in the specified area, thereby contributing positively to maximizing energy efficiency. Additionally, the proposed promising topology can be effectively applied to remotely manage diverse controllable devices. Figure 1 summarizes the proposed deep learning with the IoT structure for energy management of the air conditioner. The following points conclude the main contributions of this work:

- Introducing deep learning with IoT topology for energy management of smart buildings as an effective target for Industry 4.0.
- Proposing a new structure for the control of air conditioner operation based on IoT.
- Suggesting YOLOv3 as an advanced intelligent algorithm for people recognition.
- Controlling the air conditioner operation automatically based on the detected number of persons in a specific area instead of conventional methods.
- Publishing all events on the dashboard of the IoT platform.
- Recording all operation status of the air conditioner on the IoT database for any further analysis to manage the energy consumption of the air conditioner.
- The proposed method can be applied to other devices in order to decrease energy loss and cost.

The proposed approach is a promising tool towards the implementation, in future work in which Industry 4.0 will be fully considered, of Industry 4.0 in smart energy systems. The rest of the sections of this paper are listed as follows: Section 2 describes the proposed architecture based on IoT topology. The suggested deep learning algorithm for people recognition is presented in Section 3. Section 4 presents the experimental results and discussions. Finally, the conclusions of this work are illustrated in Section 5.

## 2. IoT Based Architecture Description

In this section, the IoT based architecture is described in detail since it is the main component of the proposed approach. Smart energy management can be utilized to decrease energy consumption in buildings, while simultaneously contributing to the comfort and security of the building. The building energy monitoring system presented in this work follows the general IoT architecture, which includes devices, connectivity, cloud, data acquisition, and application modules [23]. Multiple IoT devices of the system are equipped with electronics such as sensors and microcontrollers; they can integrate to perform a diverse set of processes. Specifically, sensors are used to sense the environment, then the collected data are transferred to the cloud through gateways for further computing. The data are analyzed in real-time and give the user complete control over the decision-making process. Once the decision is taken, then corresponding feedback is transmitted to the microcontroller on the system in order to turn on/off the units accordingly. An example of smart buildings that are interconnected to electrical power systems is illustrated in Figure 1a. Further, an IoT architecture for monitoring energy consumption is presented in Figure 1b, where the air conditioner control units inside these buildings are connected remotely to CONTACT Elements of the IoT platform. This platform allows the system to monitor devices in real-time. Data acquisition can be conducted through interfaces, such as the use of open platform communications (OPC), Modbus, and the message queuing telemetry transport (MQTT) network protocol. Although the data collection from devices can be implemented with various IoT platforms through edge computing and IoT cloud getaways, real-time process monitoring may be reflected by the choice of data acquisition relevant to the selection of relevant features, scaling and data filtering, data resampling, and dimensionality reduction of data [24]. An appropriate method, in each case, can be chosen depending on the nature of the process phenomenon. In this scheme, the CONTACT Elements for IoT@ [25], a complete platform from edge connectivity to business applications for customers via the web using a Digital Twin, is deployed to quickly evaluate the collected data and monitoring the devices intelligently. The CONTACT Elements for IoT uses standard MQTT protocols to visualize such information through a graphical dashboard after being processed by various signal processing and machine learning techniques.

## 3. YOLO Algorithms

YOLO is known as a convolutional neural network (CNN)-based object detection network and an effective algorithm for online object recognition in the deep learning field. It was first established by J. Redmon et al. [26]. The procedure of the YOLO algorithm is that a unique neural network utilizes full images directly in one computing process to extract bounding boxes and confidences for multiple categories. The YOLO network directly performs regression, which can detect targets in the image without the requirement of the regional proposal network. This allows YOLO to perform extremely fast detection. It can effectively define and encode contextual information for all classes during the training and testing process. In addition, YOLO recognizes the generalizable features of objects. Therefore, this algorithm can be integrated with the most innovative techniques in the computer vision process like region-based CNN (R-CNN) and single-shot detection (SSD).

In the YOLO algorithm, the vision process is started by dividing the input image into an (*S* × *S*) net. Then, the predicted object is centralized in the net cell. Each net cell estimates *N* bounding boxes and calculates their confidence score. The confidence score depends on the probability of the estimated box capturing an object of *Pr(object)* and the performance of the predicted box by computing intersection over union $IoU_{pred}^{truth}$. Thus, confidence scores are defined as $Pr*IoU_{pred}^{truth}$, where:

(1) $$Pr=\{\begin{array}{l} 1\;if\;the\;object\;exit,\;the\;confidence\;score=IoU_{pred}^{truth}\\ 0\;otherwise \end{array}$$

The center of objects can be detected for each grid cell by predicting one group of class probabilities, $C=Pr(Class_{i}|Object)$, regardless of the number of *N* boxes. It is assumed that the contribution from the grid cell is only determined if it contains an object. 

Each predicted box consists of five components (*x*, *y*, *a*, *b*, *confidence*), in which (*x*, *y*) describes the center of the box according to the corresponding grid cell. *a* and *b* stand for the weight and height of the entire image. The normalization of these four coordinates (*x*, *y*, *a*, *b)* is rescaled to [0, 1]. At testing time, the class-specific confidence score of every box with its respective conditional class probabilities is determined by multiplying the individual box confidence estimation with the class-conditional probabilities, as shown in Equation (2).

(2) $$Pr\left(Class_{i}|Object\right)\times Pr\left(Object\right)\times IoU_{pred}^{truth}=Pr\left(Class_{i}\right)\times IoU_{pred}^{truth}$$

where both the fitness between the estimated box and the target and the evaluated probability of class-specific targets in the box are taken into account. During the YOLO training process, the loss function is formulated by following Equation (3), where $P_{i}$ represents the confidence scores, ${𝕝}_{ij}^{obj}$ shows the existence of objects and the prediction that is determined by the *j*th bounding box predictor. The stability of the training is controlled by two parameters, $\sigma_{coord}$ and $\sigma_{noobj}$. The loss function of YOLOv3 is a multi-task loss function which is defined in Equation (4).

(3) $$\left\{ \begin{array}{c} L=\sigma_{coord}\overset{s^{2}}{\underset{i=0}{\sum }}\overset{N}{\underset{j=0}{\sum }}{𝕝}_{ij}^{obj}\left[{\left(x_{i}-{\hat{x}}_{i}\right)}^{2}+{\left(y_{i}-{\hat{y}}_{i}\right)}^{2}\right]+\sigma_{coord}\overset{s^{2}}{\underset{i=0}{\sum }}\overset{N}{\underset{j=0}{\sum }}{𝕝}_{ij}^{obj}\left[{\left(\sqrt{a_{i}}-\sqrt{{\hat{a}}_{i}}\right)}^{2}+{\left(\sqrt{b_{i}}-\sqrt{{\hat{b}}_{i}}\right)}^{2}\right]+\overset{s^{2}}{\underset{i=0}{\sum }}\overset{N}{\underset{j=0}{\sum }}{𝕝}_{ij}^{obj}\left[{\left(P_{i}-{\hat{P}}_{i}\right)}^{2}\right]\\ +\sigma_{noobj}\overset{s^{2}}{\underset{i=0}{\sum }}\overset{N}{\underset{j=0}{\sum }}{𝕝}_{ij}^{noobj}\left[{\left(P_{i}-{\hat{P}}_{i}\right)}^{2}\right]+\overset{s^{2}}{\underset{i=0}{\sum }}{𝕝}_{ij}^{obj}\underset{c\in classes}{\sum }{\left[p_{i}\left(c)-{\hat{p}}_{i}(c\right)\right]}^{2} \end{array}\right.$$

(4) $$\left\{ \begin{array}{c} L=\sigma_{coord}\overset{s^{2}}{\underset{i=0}{\sum }}\overset{3}{\underset{j=0}{\sum }}{𝕝}_{ij}^{obj}\left[{\left(x_{i}-{\hat{x}}_{i}\right)}^{2}+{\left(y_{i}-{\hat{y}}_{i}\right)}^{2}\right]\;+\sigma_{coord}\overset{s^{2}}{\underset{i=0}{\sum }}\overset{3}{\underset{j=0}{\sum }}{𝕝}_{ij}^{obj}\left[{\left(\sqrt{a_{i}}-\sqrt{{\hat{a}}_{i}}\right)}^{2}+{\left(\sqrt{b_{i}}-\sqrt{{\hat{b}}_{i}}\right)}^{2}\right]+\overset{s^{2}}{\underset{i=0}{\sum }}\overset{3}{\underset{j=0}{\sum }}{𝕝}_{ij}^{obj}\left[-{\hat{P}}_{i}log(P_{i})-\left(1-{\hat{P}}_{i}\right)\text{log}\left(1-P_{i}\right)\right]\;\\ +\sigma_{noobj}\overset{s^{2}}{\underset{i=0}{\sum }}\overset{3}{\underset{j=0}{\sum }}{𝕝}_{ij}^{noobj}\left[-{\hat{P}}_{i}\text{log}\left(P_{i}\right)-\left(1-{\hat{P}}_{i}\right)\text{log}\left(1-P_{i}\right)\right]+\overset{s^{2}}{\underset{i=0}{\sum }}{𝕝}_{ij}^{obj}\underset{c\in classes}{\sum }{\left[-{\hat{p}}_{i}\left(c\right)\text{log}(p_{i}\left(c))-(1-{\hat{p}}_{i}(c\right))\text{log}\left(1-p_{i}\left(c\right)\right)\right]}^{} \end{array}\right.$$

**Figure 2:**
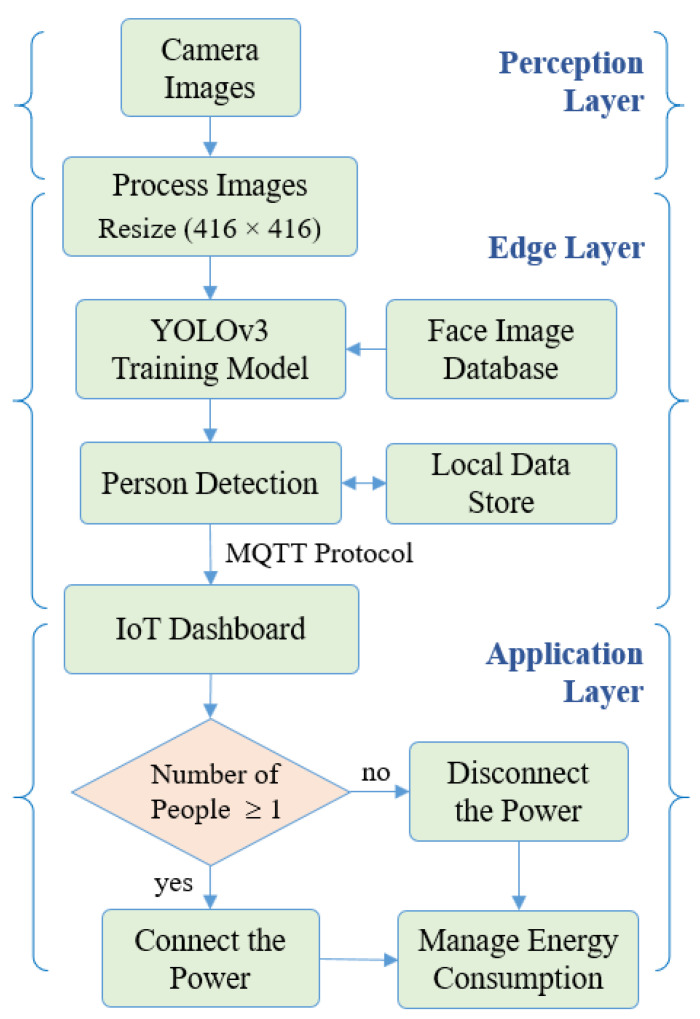
Flowchart for the solution steps of the proposed system.

**Figure 3:**
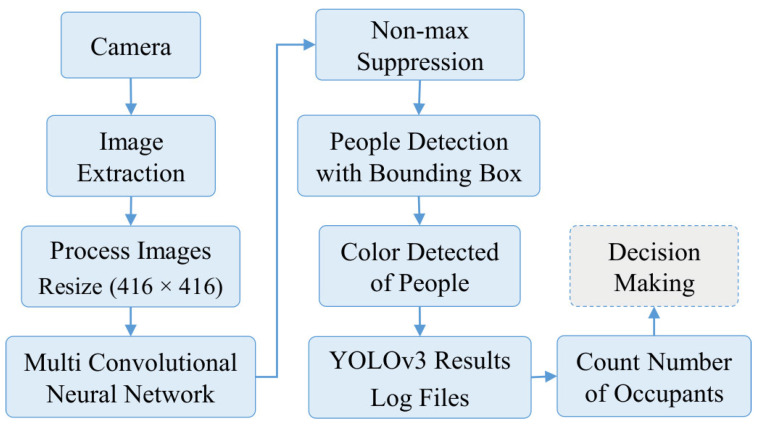
YOLOv3 algorithm outline.

**Figure 4:**
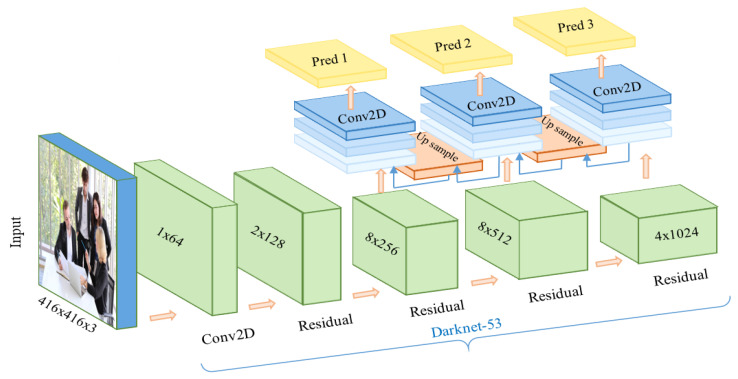
Network structure diagram of YOLOv3.

**Figure 5:**
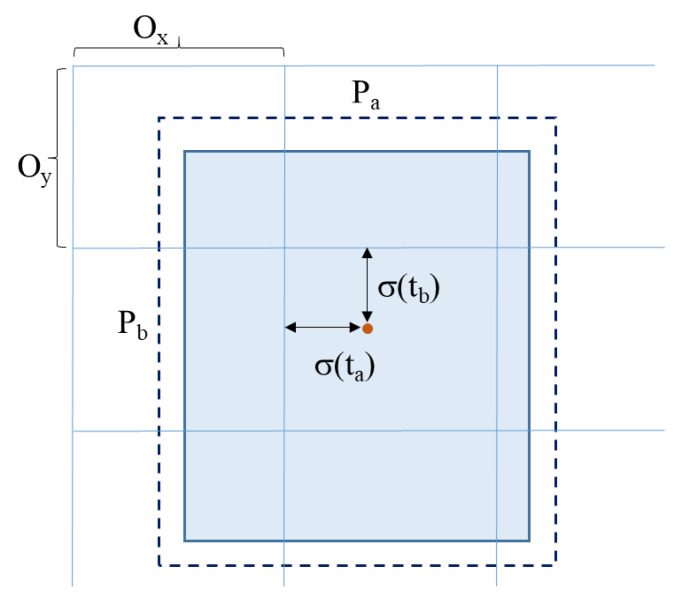
Bounding box with local prediction.

The network of YOLO originally has 24 convolution layers for feature mapping and two fully connected layers are followed to determine the coordinates of the bounding box and their respective object probabilities. The depth dimension of the feature maps is decreased by alternating 1 × 1 convolutional layers. The network can process images at 45 frames per second and a fast version of YOLO can reach 155 frames per second with less accuracy. An improved version of YOLOv2 is developed as a faster version than other recognition techniques. It can handle various image sizes to increase the speed vs. accuracy tradeoff [27]. Figure 2 shows the flowchart for the solution steps of the proposed system. The third version of the YOLO algorithm named YOLOv3 has been introduced, which is faster and more accurate than YOLOv1 and YOLOv2. The network of YOLOv3 can provide better performance on different scales by increasing its size and adding shortcut connections towards residual networks [28]. Therefore, it is able to perform complex tasks of object detection with high precision including small objects. The outline of the suggested YOLOv3 algorithm is described in Figure 3, in which the camera image is pushed into the network to estimate the output of the bounding boxes and then the people are detected. Figure 4 describes the proposed YOLOv3 network architecture representation. The feature extraction network is updated by utilizing the residual network instead of using a fully connected layer and a pooling layer. This allows the network to maintain the convergence with deeper learning and to improve the performance of training. Besides, the feature extraction is developed based on the Darknet-53 network for obtaining deeper feature information. The network prediction process starts with the input images of size 416 × 416 pushing into the Darknet-53 network with a total of 53 convolutional layers which can offer better performance [29]. Then, several convolutions with five down samplings are applied. The details of the Darknet-53 network parameters are listed in Table 1. The bottom level down-sampling feature map is 13 × 13, and the two upsampling feature maps are 26 × 26 and 52 × 52, respectively [27]. The suggested YOLOv3 network has 32 times down-sampling of the input recognition image and has a route layer for shallow feature detection. The middle layer and the fifth layer of DarkNet and the upsampling perform a double up-sampling operation then stitch them onto the feature map. YOLOv3 provides three outputs of feature maps. The feature maps of various sizes are evaluated for the recognition of small objects. Therefore, it is able to recognize relatively large-sized targets in an image [25,30,31,32]. Finally, predictions of bounding boxes for each cell on the feature map are carried out in the network output using Equations (5)–(8), where the center coordinates and size of the obtained bounding box are described by Bx, By, Ba, Bb, respectively, as seen in Figure 5. Four coordinates (tx, ty, ta, tb) of bounding boxes are estimated using YOLOv3. O_x_ and O_y_ describe the offset of the cell. P_a_ and P_b_ are the width and height of the bounding box before prediction.

(5) $$B_{x}=\sigma \left(t_{x}\right)+O_{x}$$

(6) $$B_{y}=\sigma \left(t_{y}\right)+O_{y}$$

(7) $$B_{a}=P_{a}e^{t_{a}}$$

(8) $$B_{b}=P_{b}e^{t_{b}}$$

## 4. Results and Discussion

**Figure 6:**
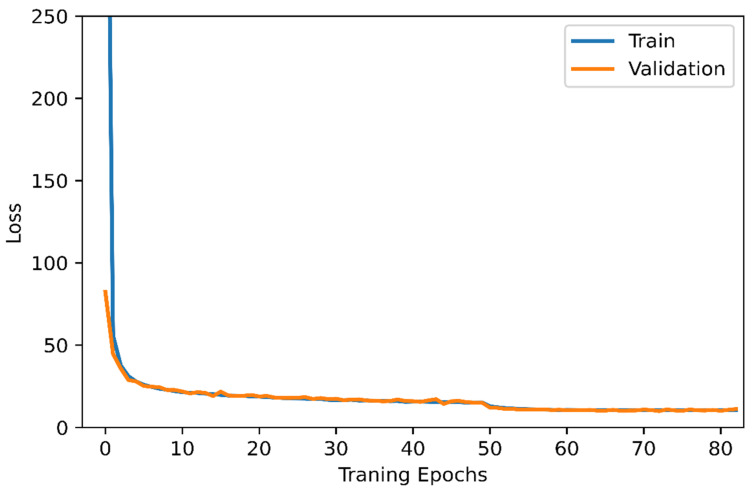
The training effort of the proposed YOLOv3 to develop the deep learning model.

**Figure 7:**
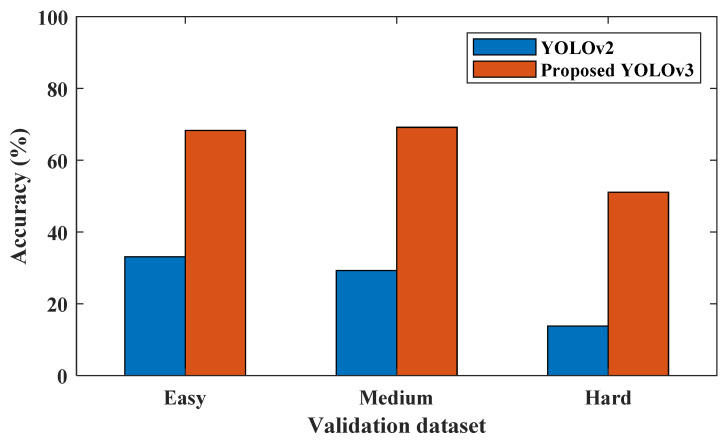
The accuracy of different YOLO algorithms in the case of easy, medium, and hard validation datasets.

**Figure 8:**
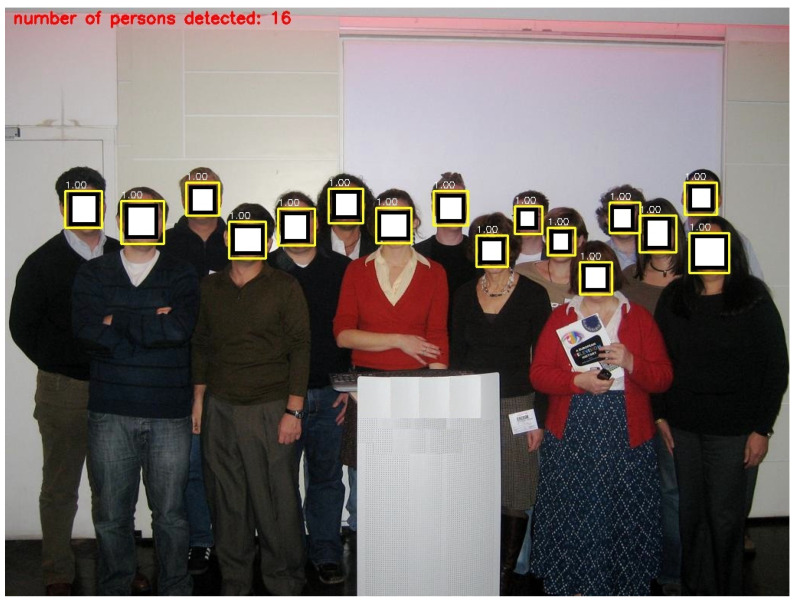
The output result of the proposed YOLOv3, which aimed to recognize a large number of persons (released from WIDER dataset [34]).

In this section, the deep learning architecture is carried out based on the YOLOv3 algorithm to detect the number of persons in a specific area. In this study, the deep learning-based people detection utilizing the YOLOv3 algorithm is performed to count the number of persons in a specific area to manage the operation of the air conditioners for energy efficiency. Firstly, the suggested YOLOv3 model is trained using the WiderFace data set, which is known as a face detection benchmark dataset, and selected from the publicly available wider dataset, which contains 32,203 images and 393,703 labelled face images. The log information on each iteration of the training model was collected. The performance of YOLOv3 model is examined by the loss function in Equation (4). Figure 6 illustrates the loss function of the training and validation set; both of them are steadily decreased and tend to coincide after 83 epochs. Furthermore, Figure 7 shows the accuracy of different YOLO algorithms in the case of easy, medium, and hard validation datasets. This figure shows that the proposed YOLOv3 has high accuracy compared with the YOLOv2 in the case of different validation datasets [33]. Furthermore, the proposed YOLOv3 is tested with a sample photo to confirm the capability of the model to detect a large number of persons (16 persons) before the real-time implementation. Figure 8 shows that the proposed YOLOv3 can detect all numbers of persons in the sample photo without any error, where this original photo is taken from the WIDER dataset [34]. Then, the model can be used for testing the face detection in real-time with the camera. A number of people are counted and sent to the IoT broker via the MQTT protocol. Besides, the status of air conditioners status is detected by the microcontroller. Then, the status of the air conditioner is sent via the MQTT protocol to the IoT broker. The gateway undertakes people recognition, then it publishes the number of persons to the IoT broker. The IoT platform compares the number of persons and the status of the air conditioner to make the decision. Figure 5 shows the closed-loop control circuit of the air conditioner operation. This circuit conducts automatic operation for the air conditioner based on the number of persons in a specific area. Furthermore, the circuit is provided by a manual operation as a backup system instead of the automatic operation to continue the air conditioner operation if the automatic circuit encounters any problems.

**Figure 9:**
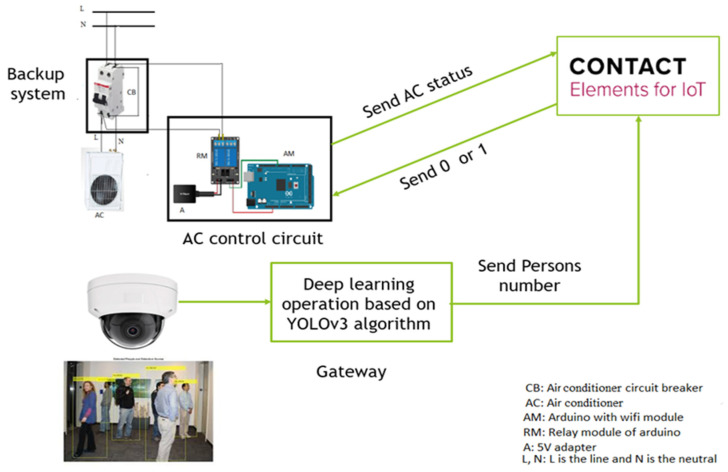
Air conditioner energy control circuit based on deep learning and IoT.

The previous circuit in Figure 9 controls the air conditioner operation as follows:

- The camera detects people in the specified area and the gateway detects the number of persons by utilizing the YOLOv3 algorithm, then it sends the number of persons via the MQTT protocol to the IoT platform.
- If there is no person in the specified area, the IoT platform will send a “0” signal after a certain time delay, via the MQTT protocol, to the microcontroller, in order to disconnect the power of the air conditioner. The air conditioner is turned off after a certain time delay, because the persons in the specified area may have left the specified area to complete a task, before returning to the specified area of the air conditioner.
- If there are some people in the room, the IoT platform will send a “1” signal by wifi to the Arduino to connect the power of the air condition, but the person must use the remote of the air conditioner to operate it, because the operation of the air conditioner is optional for people.

Noticing that the camera must capture all regions in the specified area, the test operation is carried out in a 3 × 6 m^2^ area. This challenge represents the main limitation of our method. So, a suitable camera must be selected before the implementation of the proposed energy management strategy. Furthermore, if there is any issue in the automatic control method due to the disconnection of the internet, our IoT system is provided with a backup system to continue the operation of the air conditioner, as shown in Figure 9. The final results of counting the number of persons and air conditioner operation will be recorded on the database server and presented on the dashboard of the IoT platform. The following pseudocode (Algorithm 1) summarizes the steps of the proposed IoT system.
**Algorithm 1 The Pseudo-Code of Air Conditioner Energy Control Based on IoT****1: *Capture*** the image by the camera**2: *Resize*** the image**3: *Input*** the image data to the YOLOv3 model**4: *Do*** the recognition process by the YOLOv3**5: *Publish*** the number of detected persons by MQTT protocol**6: *Do*** data analysis by Contact elements for IoT**7:  if** the number of persons ≥ 1**8:   *Send*** signal “1” by MQTT protocol to the air conditioner microcontroller***9:   Connect*** the power of the air conditioner***10:   Present*** the number of persons on the IoT dashboard**11:   *Record*** event of air-conditioned “on”**12:  *else******13:   Send*** signal “0” by MQTT protocol to the air conditioner microcontroller**14:   *Disconnect*** the power of the air conditioner***15:   Present*** the number of persons on the IoT dashboard***16:   Record*** event of the turn off the air conditioner **17:**  end if**18: *Record*** all events and data on the system database for any further analysis 

### 4.1. Scenario 1: Operation of Air Conditioner

**Figure 10:**
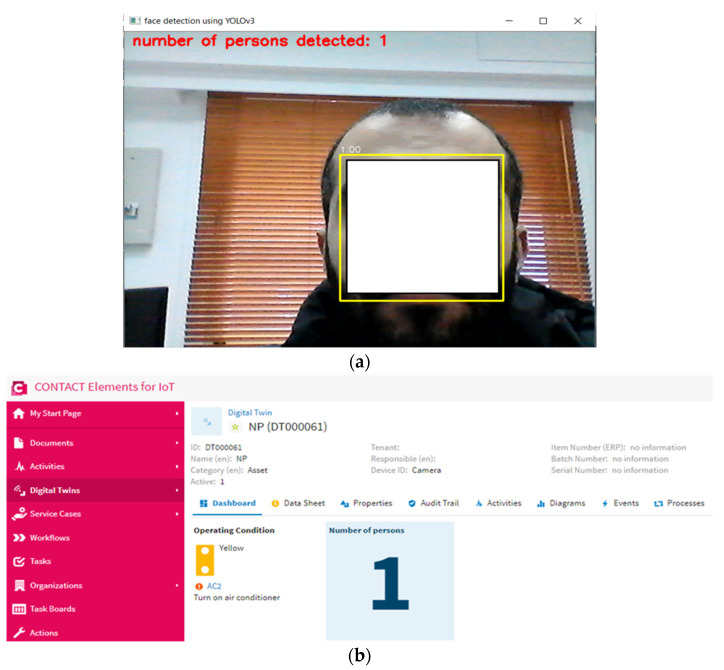
The number of persons detected in the case of scenario 1: (**a**) the output of YOLOv3 algorithm, (**b**) the presented output on the dashboard of the IoT platform.

**Figure 11:**
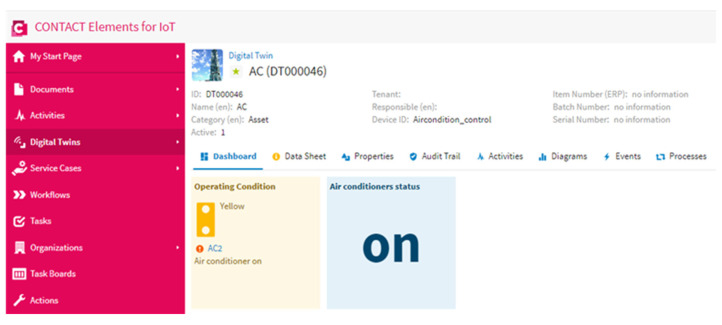
Air conditioner status in the case of detecting persons in a specific area.

In this scenario, the proposed YOLOv3 algorithm detects the number of persons in the specified area and sends the number of persons to the IoT platform via the MQTT protocol. The IoT platform analyzes the signal of the number of persons; if the number of persons is higher than “0”, the IoT platform sends a “1” signal to the microcontroller. When the microcontroller receives a “1” signal from the IoT platform, it connects the power to the air conditioner and enables any person in the specified area to operate the air conditioner. Figure 10 shows the number of persons that were detected by the YOLOv3 algorithm and presented on the dashboard of the IoT platform, while Figure 11 shows the status of the air conditioner. Figure 10 and Figure 11 show that when the number of persons is higher than “0”, the air conditioner status is on. This result concludes that the proposed IoT system works well and turns on the power of the air conditioner when the YOLOv3 algorithm detects persons in the specified area.

### 4.2. Scenario 2: Increase the Number of Persons

**Figure 12:**
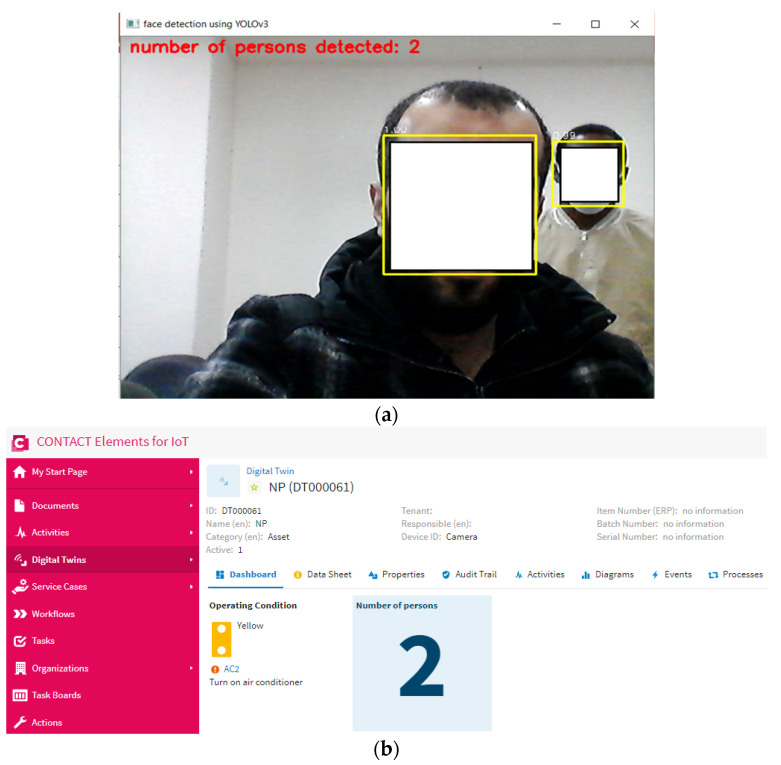
The number of persons detected—in this case, 2 persons: (**a**) the output of YOLOv3 algorithm, (**b**) the presented output on the dashboard of the IoT platform.

**Figure 13:**
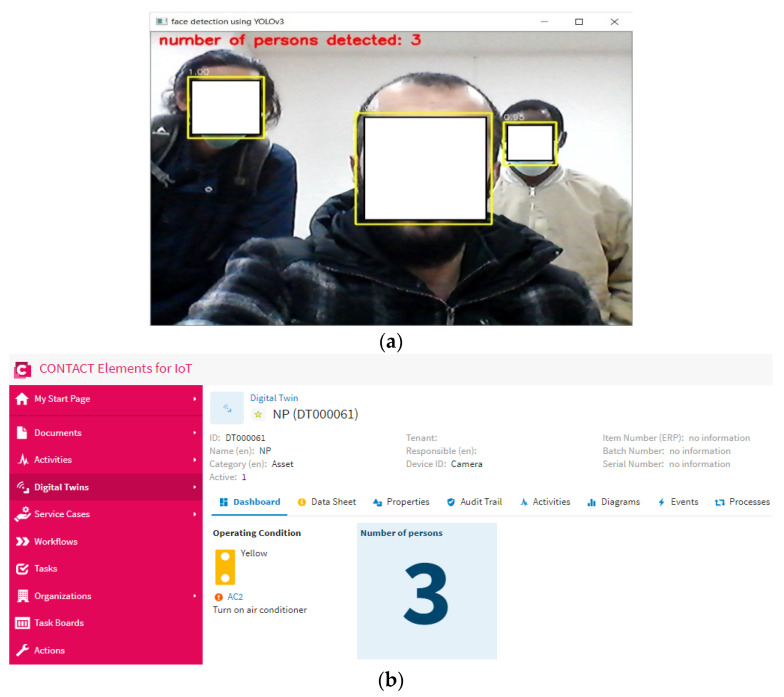
The number of persons detected—in this case, 3 persons: (**a**) the output of YOLOv3 algorithm, (**b**) the presented output on the dashboard of the IoT platform.

**Figure 14:**
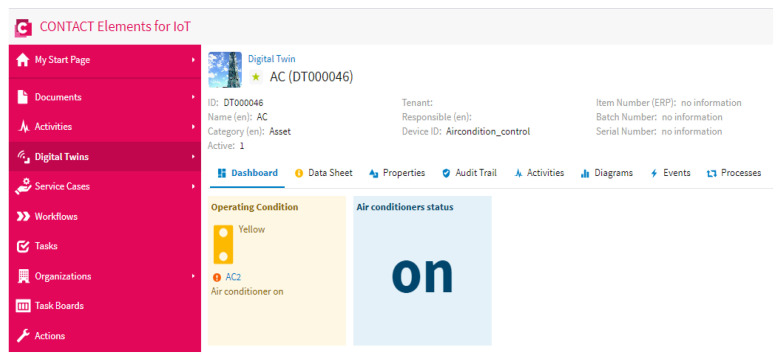
Air conditioner status in the case of an increasing number of persons being present in the specified area.

This scenario is carried out by increasing the number of persons in the specified area. In this scenario, the proposed YOLOv3 algorithm can detect all people in the specified area and send the number of persons to the IoT platform via the MQTT protocol, as shown in Figure 12 and Figure 13. Then, if the IoT finds that the number of persons is higher than “0”, it will keep the air conditioner on, as shown in Figure 14. So, the proposed IoT system can keep the air conditioner on when the number of persons increases in the specified area.

### 4.3. Scenario 3: Disconnecting the Power from the Air Conditioner

**Figure 15:**
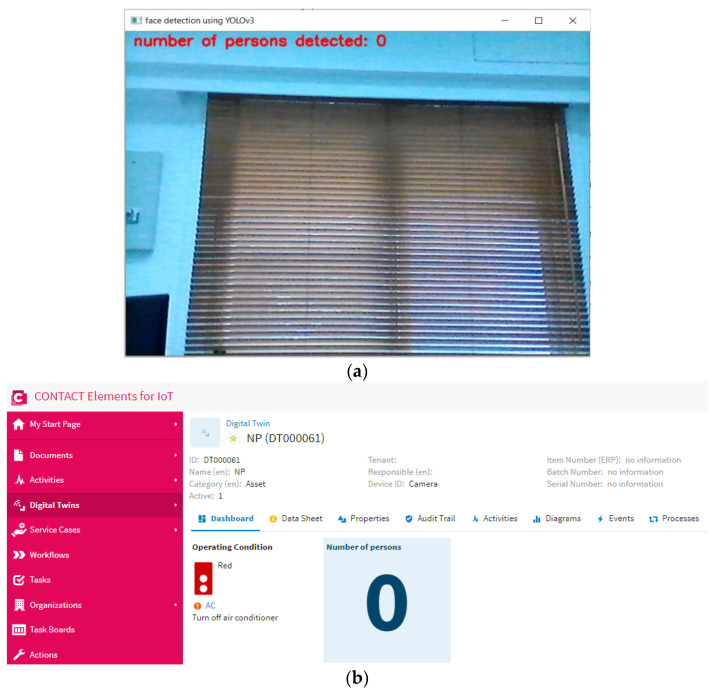
The number of persons detected in the case of scenario 3: (**a**) the output of YOLOv3 algorithm, (**b**) the presented output on the dashboard of the IoT platform.

**Figure 16:**
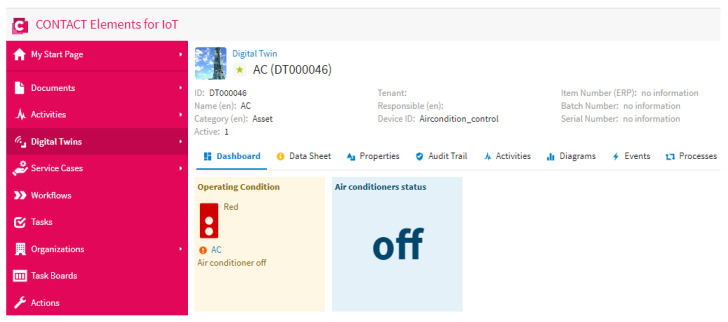
Air conditioner status in the case of no persons being present in the specified area.

This scenario is performed to test the proposed IoT system in the case of no persons in the specified area. Figure 15 shows that the proposed YOLOv3 algorithm can detect that there are no persons in the specified area and send the number of persons equal to “0” to the IoT platform via the MQTT protocol. Then, the IoT finds that the number of persons equals “0”. In this case, the IoT platform sends a “0” signal to the microcontroller of the air conditioner in order to disconnect the power of the air conditioner, as shown in Figure 16. This result concludes that the proposed IoT system can disconnect the power of the air conditioner if there are no persons in a specific area. This IoT system can decrease the cost and energy consumption due to the air conditioner operation. In the future, the proposed approach will involve detailed modeling of IoT and smart energy systems, and it will also be applied in robotics applications towards the development of industry 4.0 [35,36,37,38,39,40].

## 5. Conclusions

This paper has introduced a new IoT topology for the energy control of air conditioner systems in smart buildings based on deep learning. The proposed deep learning is carried out based on the YOLOv3 advanced recognition algorithm. The proposed IoT system controls the operation of the air conditioners based on the detection of persons in a specific area in order to decrease the cost and energy consumption due to air conditioners. Three test scenarios with different numbers of persons are created to confirm the effectiveness of the proposed IoT system. Furthermore, the proposed YOLOv3 is tested with a sample photo to confirm the capability of the model to detect a large number of persons before real-time implementation. The proposed YOLOv3 can detect all numbers of persons in the sample photo accurately. The results emphasize that the recognition algorithm can detect the number of persons in the specified area and publish it on the dashboard of the IoT platform. The proposed IoT system works well and turns on the power of the air conditioner when the YOLOv3 algorithm detects persons in the specified area. Furthermore, the IoT system can disconnect the power from the air conditioner automatically if there are no persons in the specified area. This closed-loop IoT topology enhances investments in industry 4.0. In addition, the proposed IoT system can be applied to different kinds of applications that depend on recognition and control in future work. Moreover, the proposed method will be applied to other devices in order to decrease energy loss and cost in smart grids.

## Figures and Tables

**Table 1:** Darknet-53 network structure.

	Type	Number of Filters	Size	Stride	Output
	Convolutional	32	3 × 3	1	256 × 256
	Convolutional	64	3 × 3	2	128 × 128
1×	Convolutional	32	1 × 1	1	
	Convolutional	64	3 × 3	1	
	Residual	---	---	---	128 × 128
	Convolutional	128	3 × 3	2	64 × 64
2×	Convolutional	64	1 × 1	1	
	Convolutional	128	3 × 3	1	
	Residual	---	---	---	64 × 64
	Convolutional	256	3 × 3	2	32 × 32
8×	Convolutional	128	1 × 1	1	
	Convolutional	256	3 × 3	1	
	Residual	---	---	---	32 × 32
	Convolutional	512	3 × 3	2	16 × 16
8×	Convolutional	256	1 × 1	1	
	Convolutional	512	3 × 3	1	
	Residual	---	---	---	16 × 16
	Convolutional	1024	3 × 3	2	8 × 8
4×	Convolutional	512	1 × 1	1	
	Convolutional	1024	3 × 3	1	
	Residual	---	---	---	8 × 8
	Avgpool Connected Softmax	Global 1000			

## Data Availability

The data presented in this study are available on request from the corresponding author.

## Abbreviations

MQTT	message queuing telemetry transport
IoT	internet of things
IOU	Intersection Over Union
YOLO	You Look Only Once
*B_x_, B_y_*	center coordinates obtained bounding box
*B_a_, B_b_*	size of the obtained bounding box
*C*	class probability
$C_{i}$	true confidence
${\hat{C}}_{i}$	predicted confidence
*N*	number of bounding boxes
$P_{i}$	confidence scores
*P_a_, P_b_*	the width and height of the bounding box before prediction
*P_r_*	prediction probability
*(S x S)*	number of cells of an image
*S^2^*	number of grids in the input image
*O_x_, O_y_*	the offset of the cell
${𝕝}_{ij}^{obj}$, ${𝕝}_{ij}^{noobj}$	the existence or non-existence of objects
$\sigma_{coord}$	coordinate error weight
$\sigma_{noobj}$	IoU error weight
*a, b*	box dimensions
${\hat{a}}_{i},\;{\hat{b}}_{i}$	the width and height of the predicted bounding box
*c*	the specified class related to the detected object
*(t_x_, t_y_, t_a_, t_b_)*	coordinate prediction of bounding boxes
p	true probability of object belonging to class *c* is in grid *i*
$\hat{p}$	predicted probability value
*(x, y)*	center of the box
${\hat{x}}_{i},\;{\hat{y}}_{i}$	the coordinate position of the predicted bounding box
